# The Invisible Hand, the Visible Wound, and the Commercial Determinants of Health: Complicity of Commercial Entities and the Palestine Catastrophe

**DOI:** 10.1177/27551938261417277

**Published:** 2026-01-29

**Authors:** M. Mofizul Islam

**Affiliations:** Department of Public Health, 2080 La Trobe University Bundoora, Victoria, 3086, Melbourne, Australia

**Keywords:** commercial determinants of health, palestine, gaza, complicity, corporate power

## Abstract

People often describe the ongoing catastrophic situation in Palestine, particularly in the Gaza Strip, as a political and humanitarian crisis. However, a recent report (A/HRC/59/23) by United Nations Special Rapporteur Francesca Albanese highlights the necessity of understanding the complex commercial practices of corporations that contribute—directly or indirectly—to this catastrophe. The report reveals a critical yet often overlooked aspect of public health ethics: corporate complicity in unprecedented human suffering. This article demonstrates how commercial entities contribute to public health harms, with Palestine serving as a significant and urgent case study. Using the commercial determinants of health framework, this article argues that corporate practices—such as supplying goods and services, maintaining operations or financing actors implicated in international crimes to actors implicated in international crimes through military operations in occupied territories—can constitute complicity in serious human rights violations. These actions typically occur within legal and ethical grey areas, exacerbated by gaps in global governance, opaque corporate structures, and weak accountability mechanisms. The article advocates identifying and including complicity as a fundamental practice used by commercial entities primarily for profit. It also emphasises the need to expand research, advocacy, and regulatory oversight to address the intersection of corporate power, armed conflict, and population health.

A recent investigative report by United Nations Special Rapporteur Francesca Albanese has garnered significant international attention for its examination of human rights violations in the occupied Palestinian territories.^1^ The report outlines the catastrophic operations by the Israeli military in the Gaza Strip that have been ongoing since the brutal attacks by Hamas on 7 October 2023. Entitled “From Economy of Occupation to Economy of Genocide”, the report details how numerous corporate entities have profited from Israel's illegal occupation, as well as from practices of apartheid and acts that could amount to genocide, as indicated by the International Court of Justice.^2^ The special rapporteur has compiled a database of approximately 1000 corporate entities, highlighting that this complicity is just the tip of the iceberg.

The grave situation in Palestine in general and the Gaza Strip catastrophe in particular are often framed as political and humanitarian crises. However, Albanese's report underscores the importance of understanding the complex and layered commercial practices of large corporations that contribute directly or indirectly to the conditions that shape health, infrastructure, and human rights violations in the region. The commercial determinants of health (CDoH) framework offers a powerful lens for examination of how corporate interests, particularly profiteering behaviours, intersect with armed conflict, public health collapse, and now genocide in the Gaza Strip. The concept of CDoH has rightfully gained traction, exposing the way corporate practices that are driven by profit maximisation impact population health, often detrimentally, both directly and indirectly. This concept, which has traditionally been discussed in contexts like those involving tobacco, ultra-processed foods, alcohol, and fossil fuels becomes horrifyingly pertinent when applied to situations of armed conflict and occupation, particularly the ongoing devastation in the Gaza Strip. Here, the actions of large corporations, intertwined with state military agendas, become lethal CDoH on a catastrophic scale, transforming profit motives into instruments of suffering at a population level, mass death, and genocide.

Building on this outlook, this article aims to shed light on the role that corporate actors play in the catastrophe in Palestine and to illustrate how “complicity” serves as a key mechanism through which commercial entities can impact human health and contribute to health inequities. By focusing on the situation in the Gaza Strip and occupied territories, this article argues that complicity, whether direct or indirect, should be recognised as a fundamental practice of CDoH, capable of causing harm, while also in some instances providing potential benefits. Understanding complicity as a commercial practice offers a deeper academic insight into the CDoH framework and may help the public health community, policymakers, civil society, consumers and investors.

## Commercial Determinants of Health

**Figure 1.**
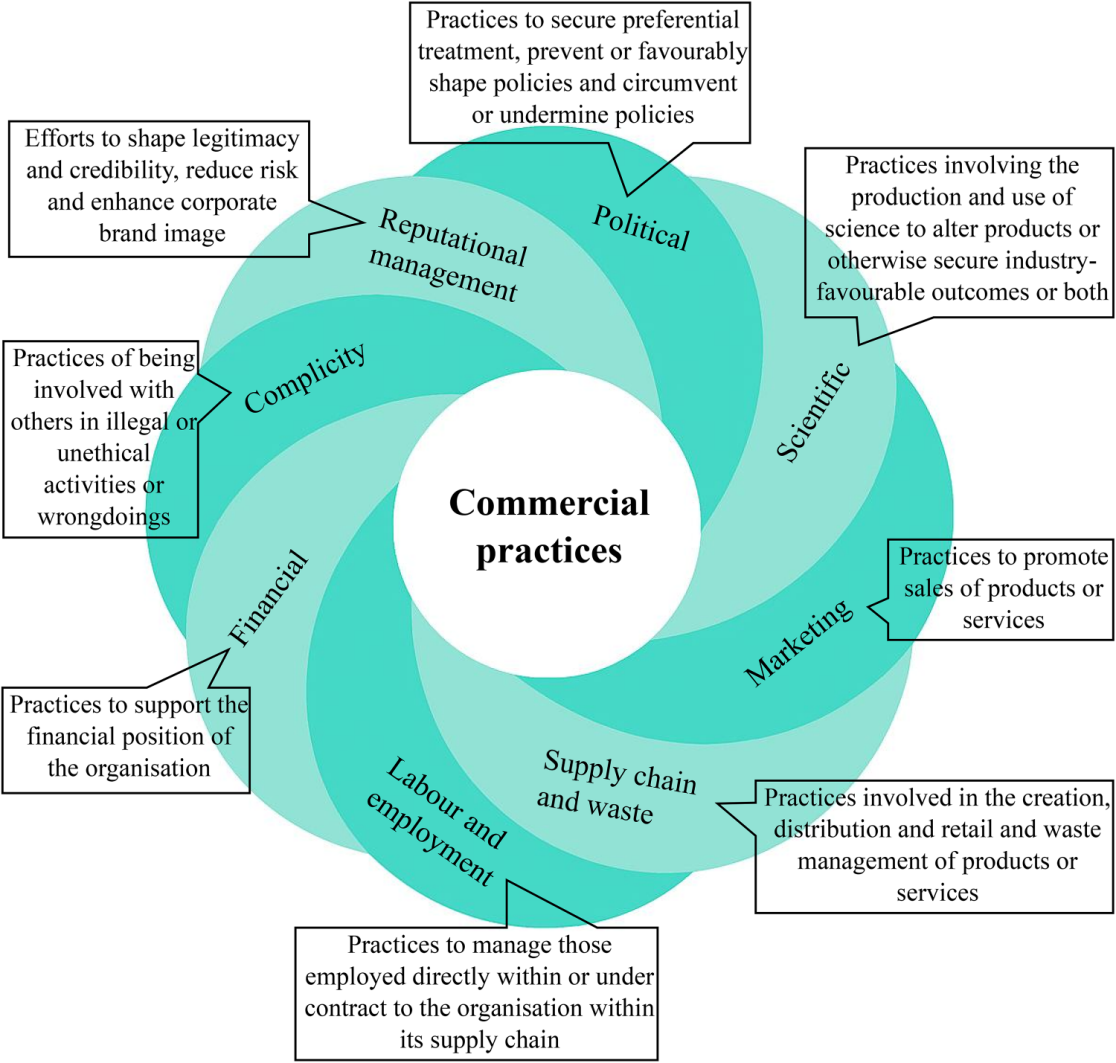
Commercial practices affecting health.

CDoH are now understood as the systems, practices, and pathways through which commercial actors drive health and equity.^3^ CDoH form a key social determinant, as the term refers to the conditions, actions, and omissions by commercial actors that affect health.^4^ CDoH are not only the products (eg, tobacco or ultra-processed food) that commercial entities produce but also the systems and, most importantly, the practices that these entities use to increase revenue. The term “system” refers to the economic, political, legal, and cultural environments in which commercial practices are embedded. Gilmore and colleagues^3^ identified seven key commercial sector practices: political, scientific, marketing, supply chain and waste, labour and employment, and financial and reputational management (see Figure 1). These practices can be overlapping and mutually reinforcing and may vary in accordance with the context in which the commercial entities operate. One of the key contributions of this framework is its ability to encourage the systematic monitoring efforts of commercial actors and their practices. The seven commercial practices mentioned above can be examined and monitored by collecting data on a range of questions. For instance, financial practice can be monitored by tracking activities such as whether the commercial entity is engaged in tax avoidance or evasion. Lacy-Nichols (2023) provided a list of guiding questions to support the application of this framework.^5^ Currently, there is a lack of systematic oversight on commercial entities and their practices. In spite of the clear evidence that some of these entities cause significant harm to health and the environment, there is currently a lack of systematic oversight on commercial entities and their practices.^6,7^ By providing a coherent conceptual foundation, the framework can aid in the development of strong indicators and accountability mechanisms designed to identify, track, and reduce harmful commercial practices.^5^

## Complicity of Commercial Entities

The term *complicity* is defined as occurring when someone is being involved with others in an illegal or unethical activity or wrongdoing.^8^ This term inherently carries a negative connotation. However, if a neutral term is needed—which may not be necessary at this stage, especially since the literature surrounding CDoH primarily addresses harms to health and the environment—the “collaboration” or “business engagement” could be used. These two terms can have positive, neutral, or negative connotations depending on the context. Commercial entities should not be complicit in activities by other actors that result in harm to health.

Like the seven commercial practices identified by Gilmore and colleagues,^3^ the activities involving complicity vary from legal or ethical to illegal or unethical, with many in the grey zone in between. For example, despite evidence from internal research showing the harmful effects of its product, a commercial entity that deliberately hides data to generate revenue may be acting legally but unethically. Complicity among the commercial entities, which is discussed by Albanese in her report, in terms of atrocities and genocide in the Gaza Strip, is both unethical and illegal. While ethical complicity is broader than legal liability and occurs more frequently, the concept of being held responsible for knowingly assisting in a wrongful act is analogous and informs ethical reasoning. The law only holds someone responsible under specific conditions. Yet, many harmful or questionable acts fall outside of what courts can prosecute.^9^ Even if the law cannot or does not punish an act, we may still see it as morally wrong. Essentially, both law and ethics rest on the same basic idea. Ethics borrows from that idea when we think about complicity; even if no law is broken, the fact that someone knowingly facilitated harm guides our moral judgment.^9^ Avoiding complicity is a responsibility to respect human rights and entails acting with due diligence to avoid knowingly contributing to human rights abuses, whether or not there is a risk of legal liability.^10^

Complicity can be seen across the value chain, which encompasses all the steps a company undergoes, from the initial idea for creating a product to its delivery to the customer, to create a good or service. This complicity can be broadly categorised as upstream, downstream, and lateral.^5,11^ Supplying artificial sweeteners, synthetic fats, or additives to ultra-processed food companies is an example of upstream complicity. A media outlet that is promoting ultra-processed foods, despite their known health impact, is involved in downstream complicity. Financing industries known to be harmful, such as tobacco and arms, even after documented abuses is lateral or institutional complicity. Another way to categorise complicity is by the degree of involvement. For example, a commercial entity that actively designs and helps to implement crimes against humanity is first-order complicit.^9^ Companies that are aware that their products or services are used to commit such crimes, yet continue their business operations, are second-order complicit. Finally, firms that derive benefits indirectly by operating within, and profiting from, a context structured by human rights violations can be seen as third-order complicit. Many of the commercial entities mentioned in Albanese's report fall into one or more of these categories and are therefore complicit.^1^

## Inclusion of Complicity as A Practice

My argument posits that we expect all commercial entities to follow the practices identified by Gilmore et al (2023) in an optimal manner that does not cause health harms. However, even if they follow the seven identified practices optimally, commercial entities still can be complicit—either directly or indirectly—with other commercial entities or actors, including regimes or states responsible for health-harming activities on a population level. Recognising this complicity is essential because the primary goal of public health, as outlined in the concepts and frameworks of the CDoH, is to identify factors, pathways, and opportunities for interventions to reduce harm.^3,5^ Ignoring this complicity would leave a significant pathway to harm to health.

Much of the corporate involvement in harm to public health is indirect, tacit, and legally ambiguous, often taking complex and opaque pathways that make accountability diffuse and difficult to enforce.^12,13^ For example, a company supplying raw materials, such as chemicals or packaging materials, to a tobacco manufacturer can be seen as an upstream actor that is complicit in the production of harmful products. However, this same company may also provide identical materials to non-harmful industries, blurring the line between routine commerce and morally questionable entanglement. If we extend the blame even further, we risk an infinite regress: should we also hold accountable those who supply raw inputs to the complicit company? Corporations often use this complexity to evade moral scrutiny and legal responsibility,^14^ arguing that because their supply chains and client bases are so diverse, they cannot be held responsible for the harmful uses of their products. By blurring the boundary between ordinary commerce and morally questionable entanglement, they can diffuse accountability and evade both public criticism and legal sanction. Nevertheless, public health frameworks, advocacy, and civil society must confront complicity—whether direct or indirect. Even in situations where commercial entities cannot be held legally liable, it is still crucial to identify and confront their complicity. Doing so promotes transparency by making the corporate practices visible and explaining how they contribute to harmful outcomes. This also helps to challenge and dismantle the structural conditions that permit the persistence of harmful commercial practices, such as weak regulation and inadequate policy measures and the consequent health harms.^12^ Recognising complicity also underscores that responsibility extends beyond compliance with the law, pressing commercial entities to uphold higher standards of ethical conduct and to adopt socially responsible business practices that protect and promote public health. Public condemnation and/or concerted global campaigns against corporate complicity may prove effective in rectifying corporate behaviours.^12^

Corporate complicity in human rights violations involves assisting or participating in gross violations of these rights, including through genocide, torture, crimes against humanity, and war crimes perpetrated by a state or state-like actors.^12^ If we direct our attention to the complicity of commercial entities in Palestine, it is clear that many are directly and/or indirectly aiding, abetting, facilitating, and contributing to Israel's acts of genocide, war crimes, and crimes against humanity.^1^ Albanese's report identified commercial entities such as Airbnb, Alphabet, Amazon, Barclays, BNP Paribas, Booking.com, Caterpillar, Chevron, HD Hyundai, IBM, Microsoft, Palantir, and Volvo,^1,15^ to name just a few, and there are many more that remain unidentified. These companies are complicit because they provide the Israeli military and settler-colonial endeavours with the infrastructure, logistical support, or financial services that sustain systems of repression and genocide. Some of these companies have been profiting from genocide, occupation, or border control technologies, while others facilitate displacement by engaging in land appropriation or infrastructure contracts in occupied territories.^1^ By obscuring responsibility and framing their roles as apolitical or purely economic, or by using the disguise that they are dealing with a sovereign country or its recognised organisations, these business entities deflect accountability.^1,15^ Hence, they contribute to the denial of self-determination and enable various structural violations in the occupied Palestinian territory.^1^

There is also a long list of related crimes and human rights violations, such as discrimination, wanton destruction, forced displacement, pillaging, extrajudicial killings, and starvation. For example, as mentioned in Albanese's report,^1^ in January 2024 Palantir announced a new strategic partnership with Israel and convened a board meeting in Tel Aviv “in solidarity”.^16^ In April 2025, Palantir's chief executive officer responded to accusations that the company's technology had contributed to the killing of Palestinians in the Gaza Strip by stating, “mostly terrorists, that's true”. The Palantir executives were aware of, and complicit in, the unlawful use of force by Israel. If commercial entities had ended their business relationships with Israel, many of these harmful outcomes could have been significantly reduced, if not entirely avoided. It is a poor excuse to argue that the products were sold to a particular country and therefore it is solely the responsibility of that country to ensure the use of the products aligns with international humanitarian laws. While it is still unclear how these large complicit corporations justify their involvement, buyers, as part of their transactions, may have to sign compliance and ethics statements or non-misuse agreements for the products, involving conditions that the products be used humanely. However, even if such paperwork exists, it does not absolve the corporations of responsibility, especially when it becomes evident that the products are being used to violate human rights.

The investigation presented in the report^1^ highlighted the extent to which commercial entities can impact our existence, health, societies, and environments. The report also provided an overview of the normative and legal frameworks governing the responsibility of the commercial entities.^1^ For instance, it refers to the UN Guiding Principles on Business and Human Rights (UNGPs) as a normative framework at the international level for the regulation of corporate conduct with respect to human rights.^17^ Two important bodies of law that apply to commercial complicity in Palestine and similar contexts are international criminal law and international humanitarian law. Under international criminal law, individuals such as corporate executives and, increasingly, corporate entities can be held criminally liable if they knowingly contribute to or profit from conflicts involving crimes against humanity.^2,18^ International humanitarian law, on the other hand, applies to non-state actors engaged in armed conflict.^19^ These laws apply to human rights violations and crimes under international law, irrespective of the actions that states do or do not take to ensure human rights. Therefore, it is an obligation for commercial entities to respect human rights even if a state in which they operate does not, and such entities may be held accountable even if they have complied with the domestic laws where they operate.^20^ After the International Court of Justice identified a credible risk of genocide in the Gaza Strip by the Israeli military^2^ and the International Criminal Court initiated proceedings for war crimes and crimes against humanity,^18^ corporate entities had a clear responsibility to refrain from any involvement and to withdraw completely and unconditionally from associated dealings.^1^ However, the continued involvement of these commercial entities, primarily for financial gain, has demonstrated how harmful the effects of complicity can be.

In 2006, the International Commission of Jurists convened an expert legal panel to identify when companies and their officials could be held legally responsible, under criminal and/or civil law, for being complicit in gross human rights abuses. The panel aimed to provide guidance on situations that prudent companies should avoid.^9^ Throughout their work, the panel identified several common responses related to causes that companies often articulate when facing allegations of complicity in such abuses (see Table 1).

**Table 1.** Some Common Excuses Commercial Entities may use to Justify Their Business Operations That Enable, Exacerbate, or Facilitate Human Rights Abuses and Their Counter Arguments.

Some common excuses for complicity	Response
We were carrying out a legitimate business activity.	The fact that a business conducts business, what in other circumstances would be a legitimate act in the ordinary course, does not absolve the company of legal responsibility if the causal link with the gross human rights abuse is established.
If we did not provide the assistance, another company would have done so and the abuses would still have occurred.	It is not a valid defence against criminal or civil liability to argue that another company would have collaborated with the main actor had the company in question not done so.
Our business is located in another country, we were nowhere near the place where the human rights abuses occurred.	A company does not need to have a physical presence in or near the location where human rights abuses are taking place to be found legally liable for complicity in those abuses, especially in our technologically advanced world with instantaneous communication.
We had no control or influence over the actions of the principal actor so why should we be blamed?	Whether a company's actions enabled, exacerbated or facilitated gross human rights abuses is always a factual question.
We were just abiding by national laws.	The company's complicity may not be considered unlawful or may not lead to legal claims in the country where it operates. However, when company representatives commit gross human rights abuses that amount to crimes under international law, they can be arrested and face criminal prosecution in many more jurisdictions than the country where the offenses occurred.
We had no choice, we were compelled to provide the assistance.	Although variations exist across different legal systems, generally, company officials must demonstrate that they were under the threat of death or serious bodily injury if they refused to carry out orders to assist in committing human rights abuses.
The principal actor involved in the human rights abuse has not been held legally responsible so how can we?	In both criminal law and the law regarding civil remedies, it is not necessary for the primary actor to be held liable before a secondary actor can be prosecuted or sued.
We are a socially responsible company and have spent a lot of money to improve the humanitarian and development well-being of the community.	Social activities by a company are irrelevant when determining whether it should be held responsible for conduct that enables, exacerbates, or facilitates gross human rights abuses. It is important to note that many companies engage in socially responsible practices as a way of managing their reputation.

There are many examples of companies and/or their officials being convicted for complicity, including the heinous crime of supplying poisonous gas to concentration camps during the Second World War.^21^ The infamous I. G. Farben trial laid the groundwork for the legal liability of corporate executives for participation in international crimes.^22^ The Lafarge case serves as a recent and significant example of corporate accountability in conflict zones.^23^ After it was revealed that the French cement company had paid millions of euros to armed groups, including ISIS, to sustain its operations at a cement plant in Syria during the civil war, the French Supreme Court upheld charges of complicity in crimes against humanity against the company. These landmark cases highlight the legal and ethical responsibilities of multinational corporations operating in high-risk environments, affirming that business decisions, even those made under pressure, do not exempt companies from accountability when they contribute to atrocities. Growing domestic and international litigations show an increasing trend in corporate accountability^24^ and highlight how corporate products and practices are intricately connected to our health and well-being.

Certain corporations today wield considerable political influence and possess economic power surpassing that of some nation-states.^25^ This asymmetry, marked by immense private power without commensurate mechanisms of public accountability, reveals a fundamental gap in global governance, and this has a substantial impact on our health and well-being. Moreover, the intricate web of corporate structures, including parent companies, subsidiaries, franchises, joint ventures, licensees, and affiliated entities, creates a complex framework that often obscures the boundaries between legal liability and legitimate business operations, irrespective of ethical considerations. This structural complexity, compounded by the interdependence among sub-entities, contractual networks, legal constructs, and operational jurisdictions, can be strategically exploited to evade accountability for harmful impacts.^14^ For example, HeidelbergCement (now Heidelberg Materials), a German multinational company that operates in the occupied West Bank through its Israeli subsidiary, Hanson Israel, was charged with direct contribution to the settlement economy. HeidelbergCement argued that the activities were managed locally by its independent subsidiary under Israeli law and therefore fell outside its direct responsibility.^26^ The situation is further aggravated by gaps in the legal and regulatory frameworks, such as the absence of binding rules in some jurisdictions, and inconsistencies, like uneven standards and enforcement across countries. Weaknesses such as these create loopholes that commercial entities can exploit by shifting harmful activities to jurisdictions that have weaker protections for the customers or by taking advantage of conflicting regulatory regimes, which can ultimately offer impunity. As a result, many harmful outcomes fall short of recognised human rights violations, even though they may clearly breach ethical standards and undermine public health. This fragmentation of legal responsibility makes accountability difficult to enforce.^13^ Nevertheless, there are reasons for cautious optimism. Growing public awareness and a slowly expanding body of research are beginning to shed light on the public health harms associated with commercial entities. Given the scale and complexity of these issues, there is an urgent need for sustained, high-quality research to improve the understanding of and to address the commercial determinants of health.

## Conclusion

The investigation presented in the report^1^ highlights the extent to which commercial entities can impact our existence, health, societies and environments. The situation in Palestine demonstrates how the visible wounds of war are shaped not only by missiles but also by the hidden hand of commerce, unregulated, unaccountable and deeply complicit. This is how the CDoH operate at their most extreme. If public health is to remain true to its ethos of justice and prevention, it must confront the political economy of harm and shed light on the pathways of complicity that link corporate behaviour to humanitarian catastrophes. Complicity resonates more profoundly in human rights contexts than in others. However, the adverse effects of complicity in non-humanitarian contexts, even if smaller in scale, can grow to be significant at the population level, given the prevalence of this practice. The dominant frameworks surrounding CDoH do not, at this time, systematically theorise complicity as a distinct practice. Incorporating complicity into the analytical framework of CDoH helps to identify methods and accomplices that lead to increased health harms while broadening the scope of accountability in global health governance.
